# Account of Diseases in an Eastern District of London, from July 20, to August 20, 1803

**Published:** 1803-09-01

**Authors:** 


### *Account of Diseases in an Eastern District of London, from July 20, to August 20, 1803.*

| | |
|---|---|
| ACUTE DISEASES. | |
| Scarlatina - - - - - - | 4 |
| Scarlatina Anginosa - | 3 |
| Rheumatismus Acutus - | 5 |
| CHRONIC DISEASES. | |
| Tussis - - - - - - - | 12 |
| Tussis cum Dyspnœa - | 6 |
| Phthisis Pulmonalis - - | 2 |
| Hydrops Pectoris - - | 5 |
| Dyspepsia - - - - - - | 8 |
| Enterodynia - - - - | 9 |
| Diarrhœa - - - - - - | 7 |
| Ascites - - - - - - - | 3 |
| Anasarca - - - - - | 5 |
| Apoplexia - - - - - - | 1 |
| Paralysis - - - - - - | 2 |
| Vertigo - - - - - - - | 2 |
| Cephalæa - - - - - - | 7 |
| Hypochondriasis - - - | 3 |
| Hæmorrhois - - - - - | 5 |
| Prolapsus Ani - - - | 1 |
| Rheumatismus Chronicus | 8 |
| PUERPERAL DISEASES. | |
| Ephemera - - - - - - | 4 |
| Dolores Post Partum - | 6 |
| Mastodynia - - - - - | 4 |
| INFANTILE DISEASES. | |
| Ophthalmia - - - - - | 2 |
| Ophthalmia Purulenta - | 1 |
| Aphthæ - - - - - - | 6 |

During the last few weeks there has been much less disease than has been experienced for a considerable time. The weather has been uncommonly fine, and, whilst we rejoice in it as peculiarly favourable to the different productions of the vegetable kingdom, and highly gratifying to the views of the husbandman, we feel no diminution of this pleasure from the fear of its appearing unfavourable in any other point of view. The animal as well as the vegetable kingdom exhibits signs of health and vigour. Although, however, it has been a season of pretty general health, there have been some instances of disease. Numerous cases cases of Scarlatina have occurred. Several of these have appeared in the simple form, unattended with any affection of the throat. The shivering by which the disease is generally introduced was slight, and the other symptoms of pyrexia were such as not to occasion any alarm. Nor in the instances of Scarlatina Anginosa did the soreness of the throat, or the fever accompanying it, put on any alarming appearance. In some of the patients, slight anasarca succeeded the other symptoms, but in others there was no appearance of this kind.

